# Joint Entropy for Space and Spatial Frequency Domains Estimated from Psychometric Functions of Achromatic Discrimination

**DOI:** 10.1371/journal.pone.0086579

**Published:** 2014-01-23

**Authors:** Vladímir de Aquino Silveira, Givago da Silva Souza, Bruno Duarte Gomes, Anderson Raiol Rodrigues, Luiz Carlos de Lima Silveira

**Affiliations:** 1 Instituto de Ciências Biológicas, Universidade Federal do Pará, Belém, Pará, Brazil; 2 Núcleo de Medicina Tropical, Universidade Federal do Pará, Belém, Pará, Brazil; Tel Aviv University, Israel

## Abstract

We used psychometric functions to estimate the joint entropy for space discrimination and spatial frequency discrimination. Space discrimination was taken as discrimination of spatial extent. Seven subjects were tested. Gábor functions comprising unidimensionalsinusoidal gratings (0.4, 2, and 10 cpd) and bidimensionalGaussian envelopes (1°) were used as reference stimuli. The experiment comprised the comparison between reference and test stimulithat differed in grating's spatial frequency or envelope's standard deviation. We tested 21 different envelope's standard deviations around the reference standard deviation to study spatial extent discrimination and 19 different grating's spatial frequencies around the reference spatial frequency to study spatial frequency discrimination. Two series of psychometric functions were obtained for 2%, 5%, 10%, and 100% stimulus contrast. The psychometric function data points for spatial extent discrimination or spatial frequency discrimination were fitted with Gaussian functions using the least square method, and the spatial extent and spatial frequency entropies were estimated from the standard deviation of these Gaussian functions. Then, joint entropy was obtained by multiplying the square root of space extent entropy times the spatial frequency entropy. We compared our results to the theoretical minimum for unidimensional Gábor functions, 1/4π or 0.0796. At low and intermediate spatial frequencies and high contrasts, joint entropy reached levels below the theoretical minimum, suggesting non-linear interactions between two or more visual mechanisms. We concluded that non-linear interactions of visual pathways, such as the M and P pathways, could explain joint entropy values below the theoretical minimum at low and intermediate spatial frequencies and high contrasts. These non-linear interactions might be at work at intermediate and high contrasts at all spatial frequencies once there was a substantial decrease in joint entropy for these stimulus conditions when contrast was raised.

## Introduction

### Visual system parallel pathways

The human visual system and the visual system of other primates comprises several separate visual pathways connecting the retina to the lateral geniculate nucleus and to the visual cortex which transmit information about blue-yellow, red-green, and achromatic aspects of a visual scene. Among them, the best studied are the P (parvocellular) and M (magnocellular) pathways [1], [2]. The M pathway transmit achromatic information at very low contrast levels and quickly saturates when contrast is raised, while the P pathway seems to perform the double duty of transmitting achromatic information at high contrast levels as well as red-green chromatic information with high sensitivity [3]–[6]. In spite of their different achromatic contrast sensitivity, there is a considerable range of contrasts that the M and P pathways are able to simultaneously respond to the appropriate stimulus[3].

In the domains of space, spatial frequency, time, and temporal frequency, the M and P pathways responses considerably overlap. It is necessary to look for the extreme of these domains to find regions where the response of one or another pathway predominates[7], [8]. It is important to understand how a couple of visual pathways that transmit information about achromatic contrast could collaborate to perform the duties of perception and action in the regions of the space, spatial frequency, time, and temporal frequency domains where their responses do overlap. Additionally, the collaboration between the M and P pathways in visual procession must occur in high contrast levels were both of them respond to visual stimuli [3].

### Discrimination of spatial extent and spatial frequency

The objective of this study was to measure psychometric functions for spatialextent discrimination and spatial frequency discrimination and then use these functions to estimate space and spatial frequency joint entropy in order to verify if, at higher levelsof the human visual system,the information provided byparallel pathwayscould combinetooptimize thevisual performancefor different tasks [9]–[12]. Discrimination in the domain of space can be studied by varying stimulus spatial position or stimulus spatial extent. In this work, spatial extent was chosen once it could be directly related to the differences in receptive field sizes of M and P cells [2]. Measurements were performed at a range of spatial frequencies and contrasts where there is evidence derived from visual evoked cortical potential (VECP) recordings that it is possible to isolate a single visual pathway by appropriate choice of stimulus spatial frequency and contrast [13]. The results can be compared with those obtained from visual stimulation that might simultaneously activate two or more parallel visual pathways. According to Souza et al. [13], the activity of a very contrast sensitive visual pathway predominates at low contrasts at all spatial frequencies. At high contrasts and intermediate and high spatial frequencies, the activity of a low contrast sensitive visual pathway predominates superposed to the activity of the high contrast sensitive visual pathway which saturates at these contrast levels.

Stimuli defined by Gábor functions [14] were used in this work once their discrimination by the visual system requires simultaneous performance in two Fourier related domains: the space domain and the space frequency domain. The essence of the method consisted in experiments performed with Gábor functions composed by unidimensional horizontal sine wave gratings and bidimensional circular Gaussian envelopes. Inaseries of measurements, the entropy in the space domain was evaluated, testing the subject's ability to discriminate stimuli that differ only in spatial extent. In a second series of measurements the entropy in the spatial frequency domain was evaluated, testing the subject's ability to discriminate stimuli that differ only in spatial frequency. The data points, representing the proportion of correct responses for each test condition, were adjusted by Gaussian functions and the spatial extent and spatial frequency entropies were estimated from the standard deviation of these Gaussian functions.The joint entropy was then estimated by multiplying the spatial frequency entropy by the square root of the space extent entropy to take in account that the stimuli comprised 1D spatial frequencies enveloped by 2D Gaussian functions. The results were then used to verify how stimulus contrast affected the joint entropy and if joint entropy remained above or equal to the theoretical minimum for unidimensional Gábor functions [14], [15]. The results suggested that at least two different visual pathways interact non-linearly at high contrasts to provide space and spatial frequency joint entropy values below the theoretical minimum. An abstract of this work was published in the ARVO Annual Meeting Abstract Book[16].

## Materials and Methods

### Ethics statement

This work was approved by the Ethical Committee for Research with Human Subjects (Comitê de Ética em Pesquisa com Seres Humanos) of the Tropical Medicine Nucleus, Federal University of Pará, Belém, Pará, Brazil; #076/2006-CEP/NMT, date of approval 28^th^ November 2006. All subjects that took part in this work gave their written consent.

### Subjects

We tested 7 adult subjects aged between 20 to 35 years old. The subjects had no history of congenital,degenerative, traumatic, toxic, or infectious diseases that could impair their visual system performance. Initial procedures comprised a routine ophthalmological exam and two additional eye tests: visual acuity measurement with Snelleno ptotypes, aiming to evaluate the ability of fine detail discrimination at high contrasts; and color discrimination with pseudoisochromatic Ishihara plates to rule out congenital color vision deficiencies of protan and deutan types. Each eye was separately tested. All individuals who participate in this study had normal visual acuity 6/6 when eye refractive state was corrected and normal trichromatic color vision.

In addition, the monocular spatial luminance contrast sensitivity of six out of seven studied subjects was measured at eleven spatial frequencies ranging from 0.2 to 30 cycles/degree of visual field following the procedure described in Rodrigues et al. [17]. The stimuli consisted of stationary, black-and-white horizontal sine-wave gratings, white coordinates u′ = 0.182 and v′ = 0.474 (Commission Internationale de L'Éclerage, CIE 1976), 43.5 cd/m^2^ mean luminance, 6.5°×5° in the visual field, placed at 3 m. Contrast threshold was found by continuously changing grating contrast until the grating was no longer visible. Each threshold estimation was repeated six times and the mean value was taken as representative of the subject's threshold.

The software for the contrast sensitivity evaluation was written in the C++ programming language and installed in two kinds of hardware. The first one consisted of an IBM POWERStation RISC 6000 (IBM Corporation, New York, New York, USA). The stimuli were generated using IBM GT4-3D graphic adapter (24 bits/8 bits per gun) and displayed on IBM 6091 19i color monitor (1280×1024 pixels, 81.32 kHz horizontal refresh rate, 77 Hz vertical frame rate). The second one consisted of an IBM-PC Pentium IV 1.7 GHz. The stimuli were generated using an Annihilator 2 graphic adapter (24 bits/8 bits per gun) (Creative Technology, Jurong East, Singapore) and displayed on a Sony Trinitron Multiscan G420 color monitor (1024×768 pixels, 98.8 kHz horizontal refresh rate, 120 Hz vertical frame rate) (Sony Corporation, Tokyo, Japan).

A dithering routine was used to obtain 10-bits gray level resolution [17].Luminance and chromaticity coordinates were measured with a CS-100A chroma meter (Konica Minolta, Mahwah, New Jersey, USA). The results obtained with a group of control subjects (n = 62, 16–30 years old) were used to estimate confidence intervals and upper and lower tolerance limits [17]–[19]. Two-tailed tolerance intervals were estimated, encompassing 90% of the population with 95% of certainty.

**Figure 1:**
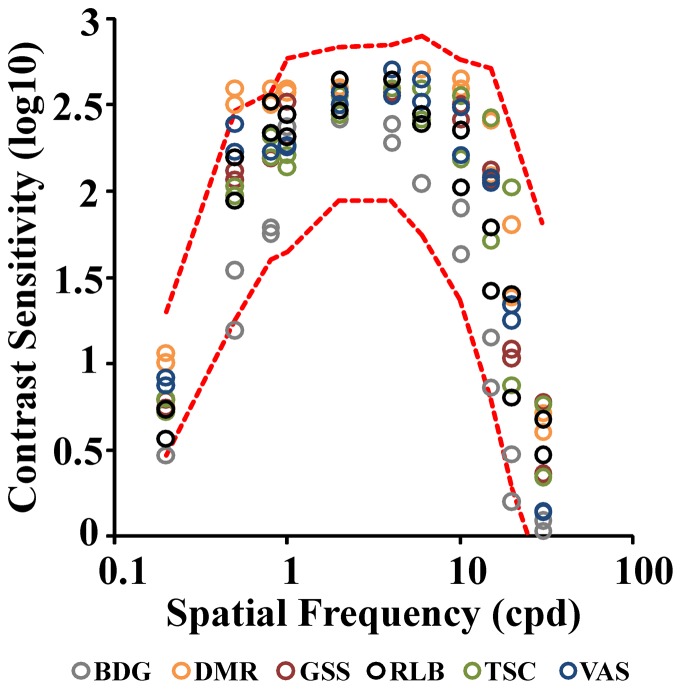
Spatial luminance contrast sensitivity for six of the seven subjects of this study. Both eyes were separately evaluated. Each data point represents either the right or left eye monocular contrast sensitivity at 11 different spatial frequencies (circles). Dashed curves represent the upper and lower tolerance limits estimated from control subjects (n = 62, 16–30 years old).

Both eyes were separately evaluated and the right and left eye contrast sensitivities (Figure 1, circles) were plotted against the upper and lower tolerance limits obtained from controls (Figure 1, dashed curves). All the six subjects had contrast sensitivities above the lower tolerance limit. It was not possible to measure the contrast sensitivities for one of the subjects of this study due to her limited time available for the experiments. However, as she passed in all the other visual exams, we decided to keep her results.

### Apparatus

The application used in this work to test spatial and spatial frequency discrimination was developed in Pascal programming language, Delphi 7 Enterprise development environment (Borland, Cupertino, California, USA) for use on IBM-PC platforms. The platform used was a Dell Precision Workstation 390, Intel Core 2 Duo 3 GHz, 2 GB of RAM, and 250 GB of hard drive (Dell,Round Rock, Texas, USA). The application controlled a stimulus generator VSG Visage model 71.02.02E (Cambridge Research Systems, Cambridge, England, United Kingdom), who designedthe stimuli on a cathode ray tube monitor Mitsubishi Diamond Pro 2070SB 20″, 800×600pixels spatial resolution, and 120 Hz temporal resolution (Mitsubishi, Tokyo, Japan).

### Luminance and chromaticity measurements

Luminance and chromaticity coordinates were measured with a ColorCal (Cambridge Research System) and the software vsgDesktop (Cambridge Research System). Throughout the experiment, stimulus mean luminance and white CIE 1931 coordinates were kept constant at 44.5 cd/m^2^and x = 0.27, y = 0.28, respectively.

### Procedure

The subjects were tested in binocular conditions and, where necessary, appropriate corrective lens were used to compensate constitutional dioptric errors.

The stimuli consisted of stationary, black-and-white unidimensionalhorizontal sine-wave gratings enveloped by bidimensionalcircular Gaussian functions. Functions of this form are called Gábor functions and their fundamental parameters are contrast, 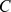, absolute phase, 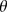, and spatial frequency of the sinusoidal frequency, 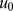, as well asextension of the Gaussian function, generally measured as standard deviation, 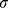:
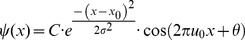
(1)The two variables that were changed in different trials were either the spatial frequency of the sine-wave grating or the standard deviation of the Gaussian envelope. Stimuli were exhibited in a 10.8° x 8.3°screen, placed at 1 m, and merged with a surround of equal mean luminance and white chromaticity coordinates.

**Figure 2:**
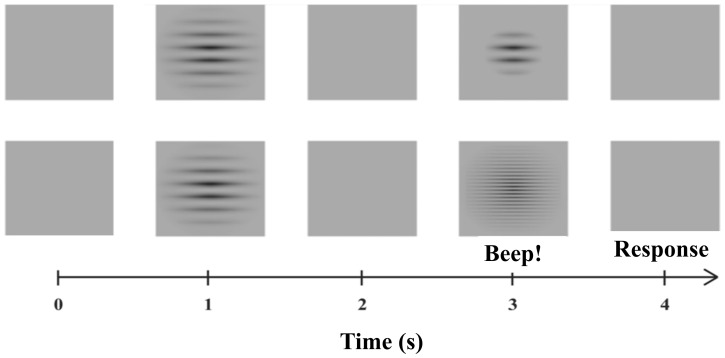
Procedure. The psychophysical measurements used a modification of the two interval forced choice task, 2IFC. A reference stimulus was compared with a test stimulus having different spatial frequency or different spatial extent. Blank fields were interleaved with reference stimulus and test stimulus. A beep indicated to the subject the moment to respond. Two forced choices were available as subject responses, either “equal stimuli” or “different stimuli”. After each trial, the spatial frequency or the spatial extent of the test stimulus was changed following the method of constant stimuli. A total of 21 different envelope's standard deviations around the reference standard deviation and 19 different grating's spatial frequencies around the reference spatial frequency were tested.

The experimentconsisted in a modification of the two interval forced choicetask (2IFC)[20], and its sequence is illustrated in Figure 2. Two stimuli were presented to the subject, interleaved with a blank screen of the same mean luminance and chromaticity, and the subject task was to answer if the stimuli were similar or different. The stimuli and blank screen were presented during 1 s each. The first stimulus presented was the reference stimulus, which always had the same standard deviation (1 degree) and spatial frequency (0.4, 2 or 10 cpd). Sequentially, an equiluminant blank field having the same chromaticity was presented to mask the previous stimulus. Further, a test stimulus was presented, which had variable standard deviation or had variable spatial frequency. After the presentation of the test stimulus, another blank field was exhibited and the subject was forced to answer if the reference and test stimuli were equal by pressing the black bottom or different by pressing the red bottom available in a CB6 control box (Cambridge Research System). We have used the method of constant stimuli to modify the stimulus parameters from trial to trial [20]. The Gábor parameters were modified at 0.05 degrees steps for spatial extent discrimination around 1 degree, as well as0.01, 0.05, and 0.25 cycles/degree for spatial frequency discrimination around 0.4, 2, and 10 cycles/degree, respectively. The percent of correct responses was recorded during the trials. We tested 21 different envelope's standard deviations around the reference standard deviation to study spatial discrimination and 19 different grating's spatial frequencies around the reference spatial frequency to study spatial frequency discrimination. Thus, for each subject, three series of psychometric functions were generated for the following parameters of the Gábor functions: 1 degree of spatial extent plus 0.4 cycles/degree of carrier spatial frequency; 1 degree of spatial extent plus 2 cycles/degree of carrier spatial frequency; 1 degree of spatial extent plus 10 cycles/degree of carrier spatial frequency. Each series comprised experiments performed at four levels of Michelson contrast: 2%, 5%, 10%, and 100%.

The data points, representing the proportion of correct responses for each test condition, were adjusted by Gaussian functions and the spatial extent and spatial frequency entropies were estimated from the standard deviation of these Gaussian functions.The joint entropy was then estimated by multiplying the spatial frequency entropy by the square root of the space entropy to take in account that the stimuli comprised 1D spatial frequencies enveloped by 2D Gaussian functions. The results were then analyzed to verify how joint entropy was affected by stimulus contrast. Particular attention was paid to verify if the joint entropy varied when the contrast was raised and if it remained above or equal to the theoretical minimum, 1/4π or 0.0796[14], [15].

## Results

**Figure 3:**
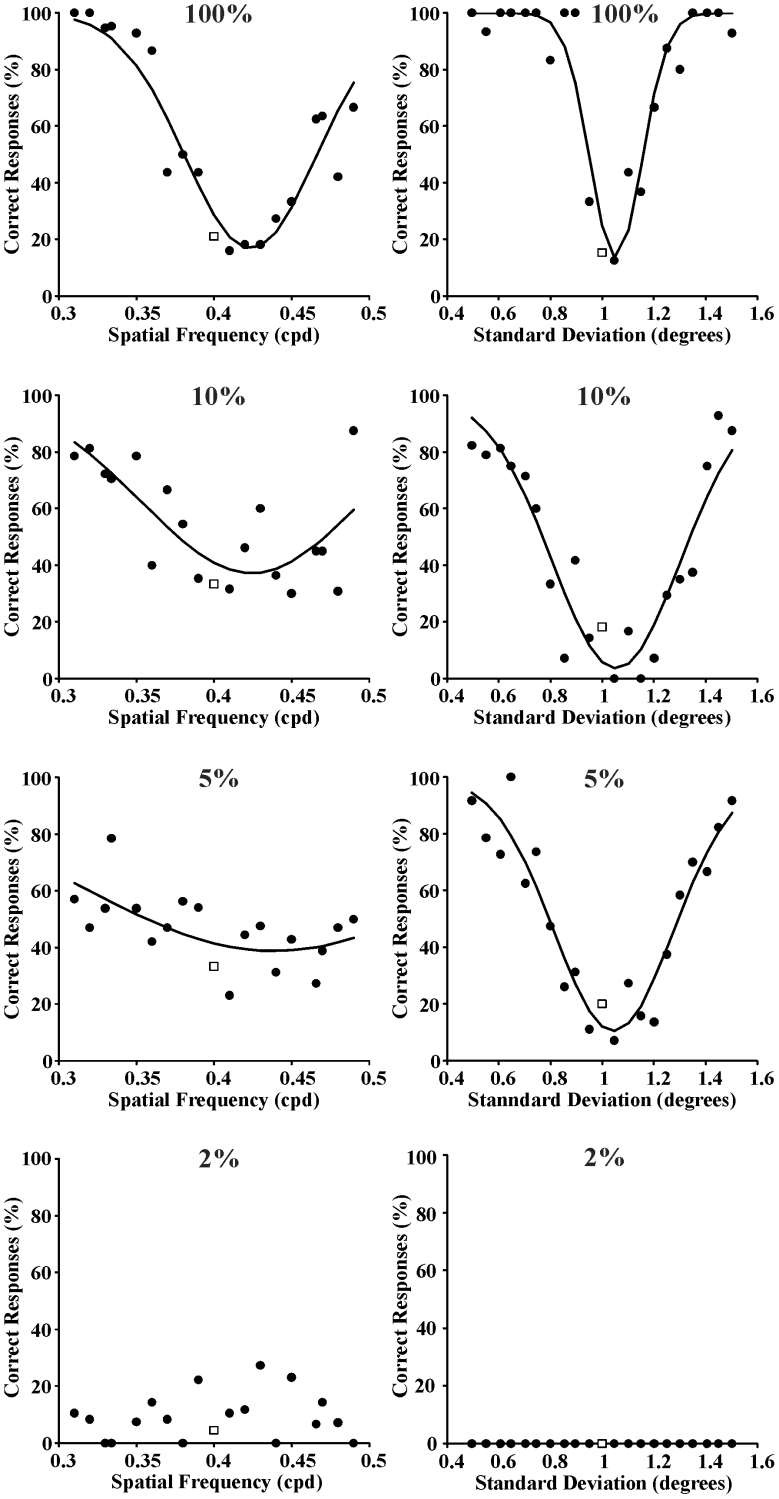
Psychometric functions obtained from Subject GSS for spatial frequency discrimination and spatialextent discrimination (left and right columns, respectively) at four different Michelson contrasts (100%, 10%, 5%, and 2% from top to bottom, respectively). Reference stimulus: 0.4 cycles/degree and 1 degree. Data points represent percent of correct responses (filled circles) or incorrect responses (empty squares). Curves are Gaussian fits to the data. The standard deviations of these Gaussian functions were used as measurement of entropy in the spatial frequency domain or spatial domain, respectively.

**Figure 4:**
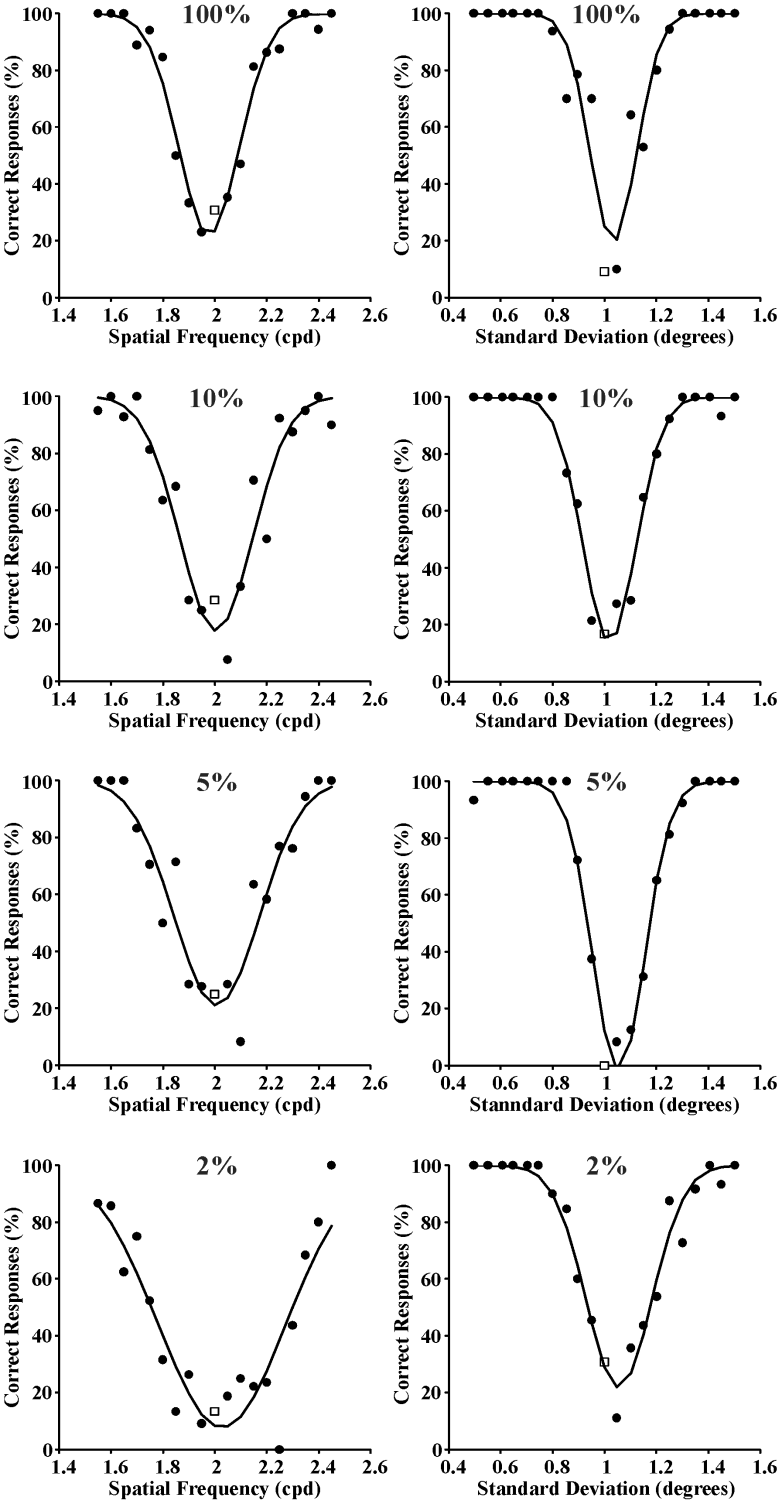
Psychometric functions obtained from Subject GSS for spatial frequency discrimination and spatial extent discrimination (left and right columns, respectively) at four different Michelson contrasts (100%, 10%, 5%, and 2% from top to bottom, respectively). Reference stimulus: 2 cycles/degree and 1 degree. Data points represent percent of correct responses (filled circles) or incorrect responses (empty squares). Curves are Gaussian fits to the data. The standard deviations of these Gaussian functions were used as measurement of entropy in the spatial frequency domain or spatial domain, respectively.

**Figure 5:**
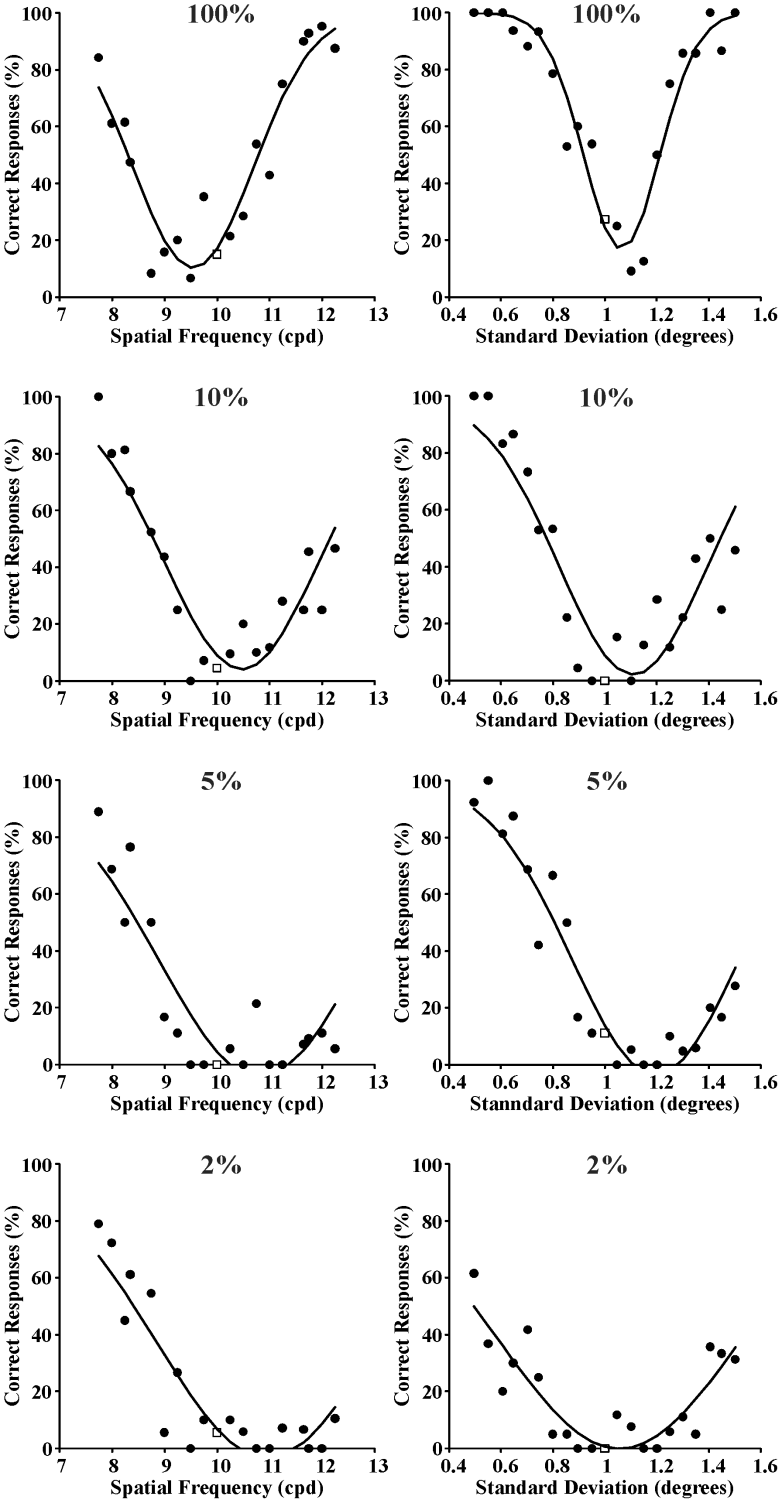
Psychometric functions obtained from Subject GSS for spatial frequency discrimination and spatial extent discrimination (left and right columns, respectively) at four different Michelson contrasts (100%, 10%, 5%, and 2% from top to bottom, respectively). Reference stimulus: 10 cycles/degree and 1 degree. Data points represent percent of correct responses (filled circles) or incorrect responses (empty squares). Curves are Gaussian fits to the data. The standard deviations of these Gaussian functions were used as measurement of entropy in the spatial frequency domain or spatial domain, respectively.

Three series of psychometric functions were obtained from each one of the seven subjects as described in the Procedure section. In addition, another three series of psychometric functions were obtained by averaging the results obtained from the seven subjects. As examples of individual psychometric functions, those obtained from Subject GSS for spatial frequency and space extent discrimination are shown in Figs. 3–5 for the following reference stimuli, respectively: 0.4 cpd and 1 degree (Figure 3); 2 cpd and 1 degree (Figure 4); 10 cpd and 1 degree (Figure 5). Data points represent percent of correct responses for each comparison between the reference stimulus and a test stimulus with equal or different spatial frequency and with equal or different spatial standard deviation (circles) or percent of incorrect responses for the comparison between two identical stimuli (squares). Curves are Gaussian fits to the data using the least square method. The Gaussian standard deviation was taken as the spatial frequency entropy or space extent entropy for each stimulus condition. From top to bottom the results obtained with different Michelson contrastsare presented: 100%, 10%, 5%, and 2%. Joint entropy (J) was estimated by the expression J = S x Fwhere S was the square root of the spatial extent entropy and F was the spatial frequency entropy, respectively.

**Figure 6:**
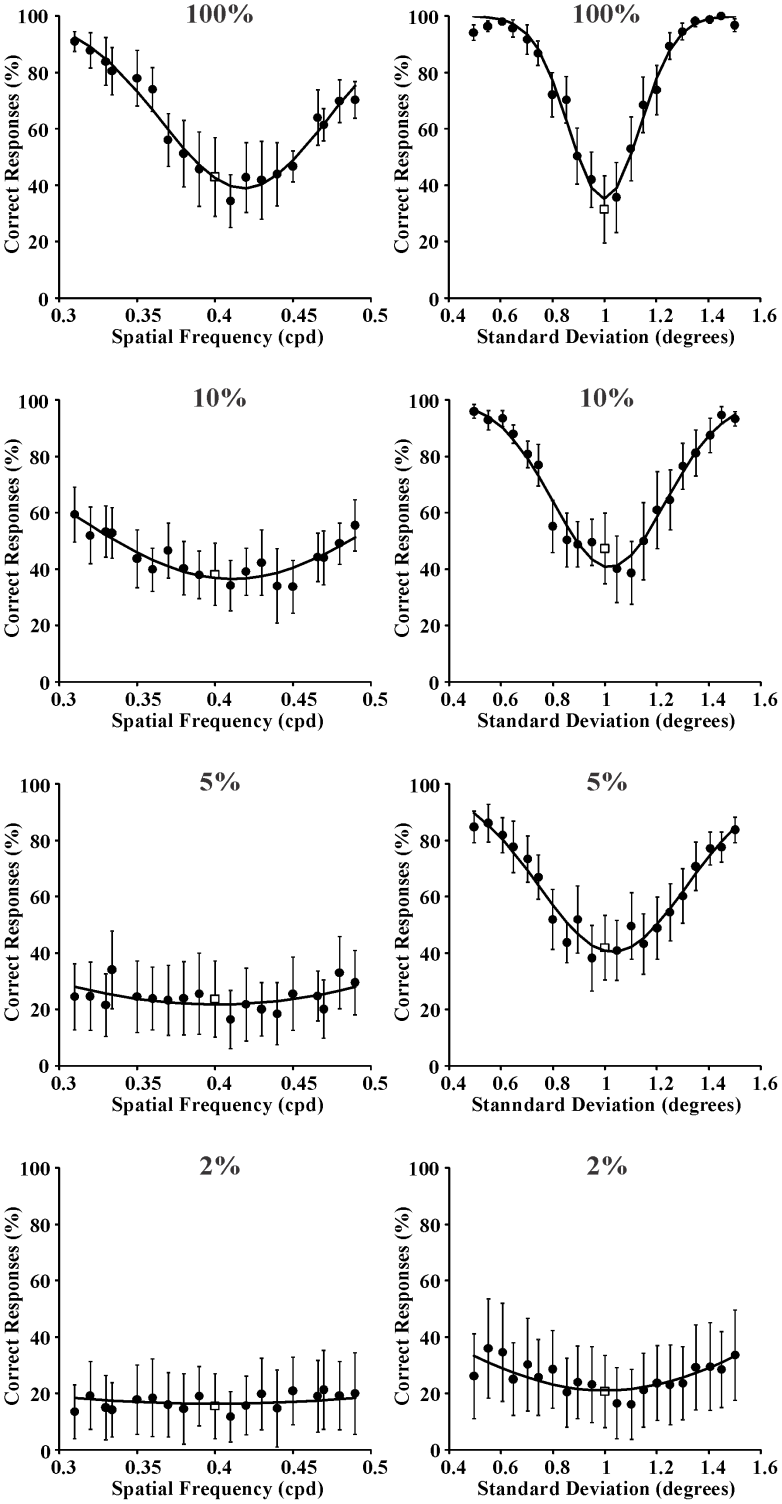
Mean psychometric functions for spatial frequency discrimination and spatialextent discrimination (left and right columns, respectively) at four different Michelson contrasts (100%, 10%, 5%, and 2% from top to bottom, respectively). Reference stimulus: 0.4 cycles/degree and 1 degree. Filled circles and empty squares represent means for correct responses and incorrect responses, respectively, obtained from the seven subjects. Vertical bars represent the standard errors of the means. Curves are Gaussian fits to the data. The standard deviations of these Gaussian functions were used as measurement of entropy in the spatial frequency domain or spatial domain, respectively.

**Figure 7:**
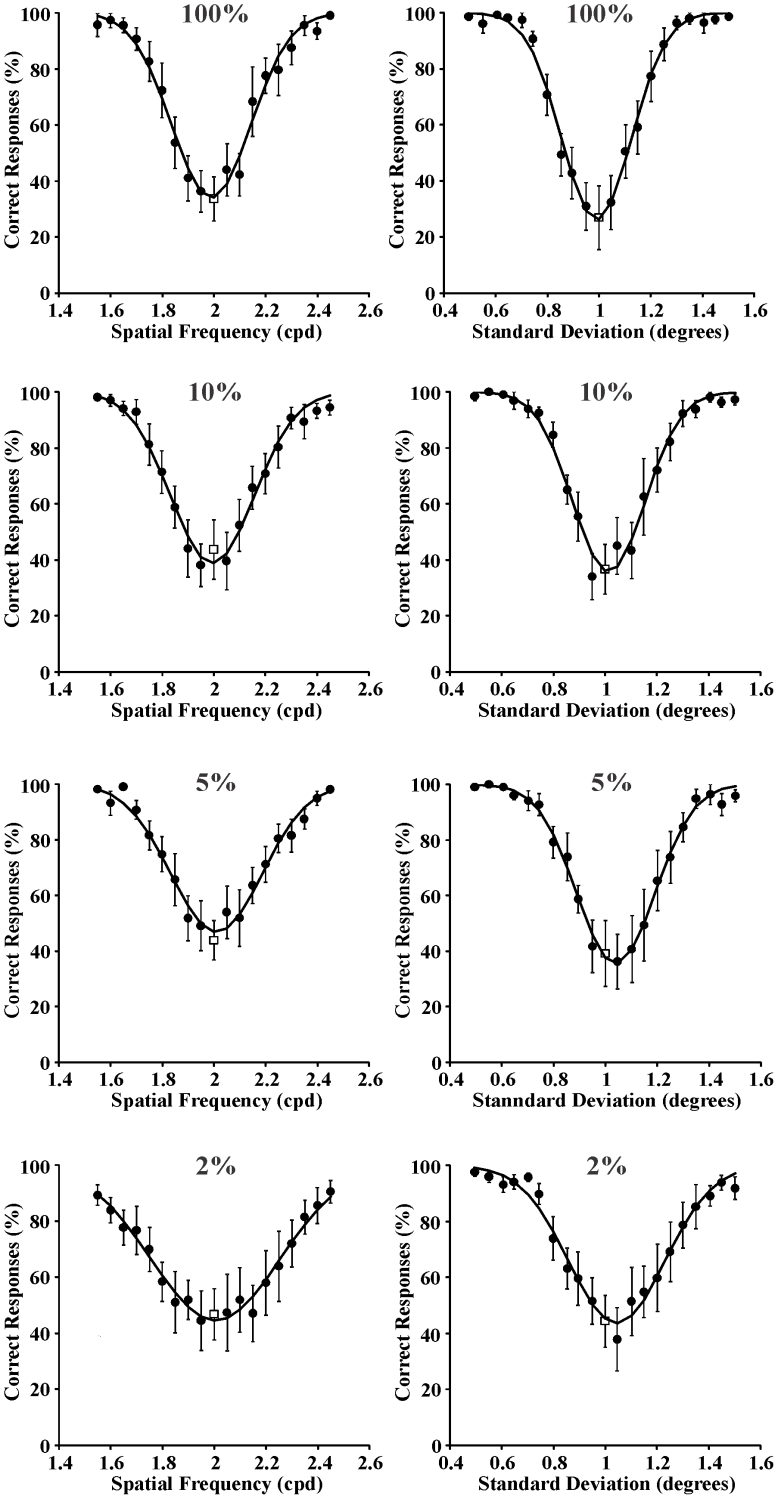
Mean psychometric functions for spatial frequency discrimination and spatial extent discrimination (left and right columns, respectively) at four different Michelson contrasts (100%, 10%, 5%, and 2% from top to bottom, respectively). Reference stimulus: 2 cycles/degree and 1 degree. Filled circles and empty squares represent means for correct responses and incorrect responses, respectively, obtained from the seven subjects. Vertical bars represent the standard errors of the means. Curves are Gaussian fits to the data. The standard deviations of these Gaussian functions were used as measurement of entropy in the spatial frequency domain or spatial domain, respectively.

**Figure 8:**
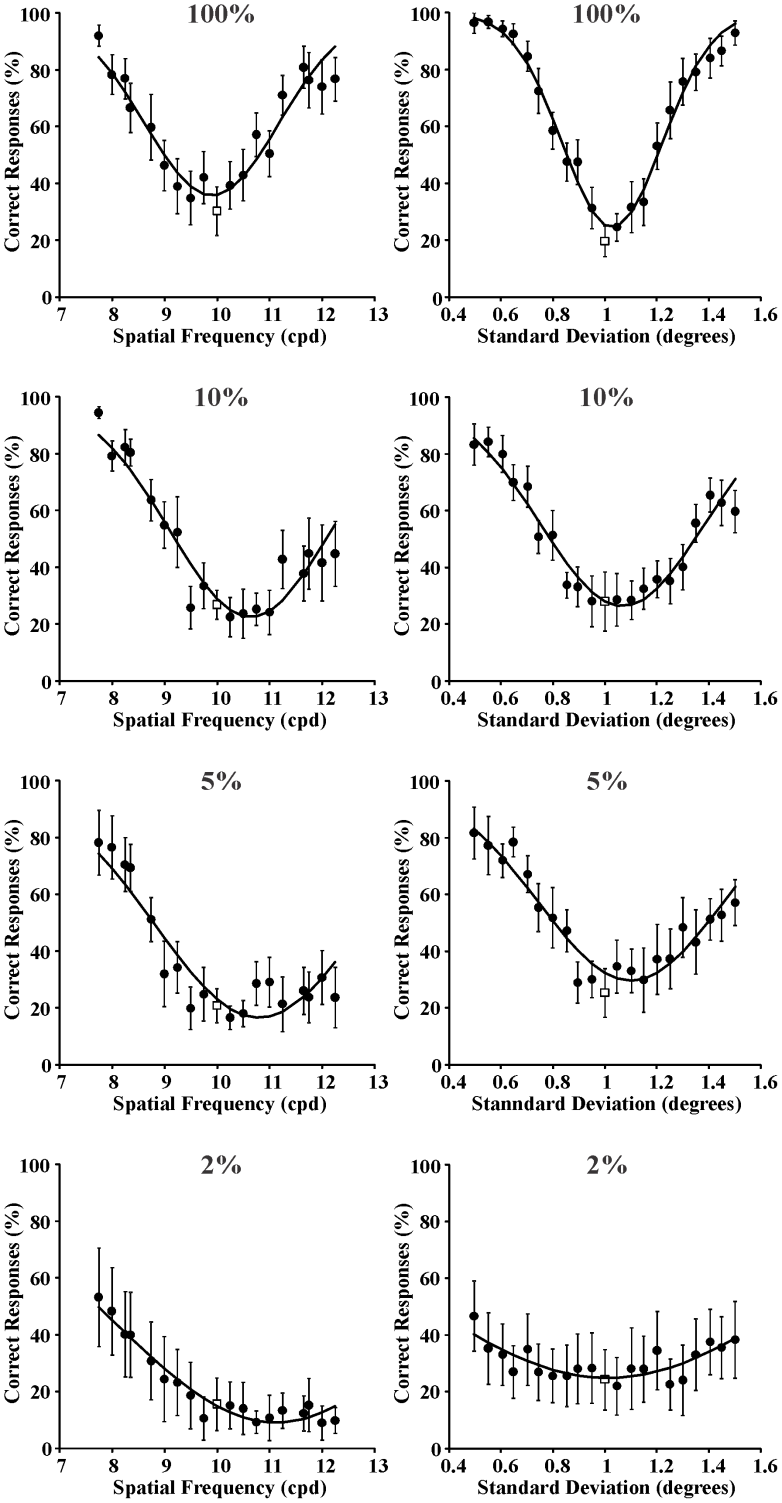
Mean psychometric functions for spatial frequency discrimination and spatial extent discrimination (left and right columns, respectively) at four different Michelson contrasts (100%, 10%, 5%, and 2% from top to bottom, respectively). Reference stimulus: 10 cycles/degree and 1 degree. Filled circles and empty squares represent means for correct responses and incorrect responses, respectively, obtained from the seven subjects. Vertical bars represent the standard errors of the means. Curves are Gaussian fits to the data. The standard deviations of these Gaussian functions were used as measurement of entropy in the spatial frequency domain or spatial domain, respectively.

Mean psychometric functions from the group of seven subjects and for spatial frequency and space discrimination are shown in Figures 6–8 for the same reference stimuli as above: 0.4 cpd and 1 degree (Figure 6); 2 cpd and 1 degree (Figure 7); 10 cpd and 1 degree (Figure 8), respectively. Data points and vertical bars represent means and standard errors for the percent of correct responses (circles) or incorrect responses (squares) at each comparison between the test stimulus and the reference stimulus. The mean values were taken as results for an “Average Subject (ASU)” and also fitted with Gaussian functions using the least square method to estimate spatial frequency entropy, spatial extent entropy, and joint entropy as described above for each individual subject.

Tables 1–3 show entropy values for spatial extent and spatial frequency estimated from the standard deviations of psychometric functions for 0.4 cpd and 1 degree (Table 1), 2 cpd and 1 degree (Table 2), and 10 cpd and 1 degree (Table 3), obtained at four different contrasts (2%, 5%, 10%, and 100% Tables 1–3 show the individual entropy values for the seven subjects, as well as entropy means and standard errors. In some cases (0.4 cpd and 10 cpd at low contrasts), it was not possible to fit Gaussian functions to the data of some individuals (empty cells in Tables 1 and 3). Tables 1–3 also show the individual values for joint entropy estimated from spatial extent entropy and spatial frequency entropy values for each subject, as well as joint entropy means and standard errors. When the value for spatial extent entropy or spatial frequency was not possible to estimate (0.4 cpd and 10 cpd at low contrasts for some subjects), it was not possible either to estimate the joint entropy (empty cells in Tables 1 and 3).

**Table 1:** Results for all seven subjects tested, means and standard errors of the means for the sample, as well as estimate results for an “average subject” (ASU; see text for details).

Subject		Contrast			
		2%	5%	10%	100%
BDG	S	0.4934	0.4972	0.4017	0.3190
	F	0.1202	0.1226	0.0703	0.0407
	J	0.0593	0.0610	0.0283	0.0130
DMR	S		0.4645	0.4019	0.2864
	F				0.0727
	J				0.0208
GSS	S		0.4813	0.4993	0.3157
	F		0.1292	0.0701	0.0424
	J		0.0622	0.0350	0.0134
IFA	S		0.6062	0.3558	0.3267
	F		0.1848	0.0914	0.0394
	J		0.1120	0.0325	0.0129
RLB	S		0.5385	0.5150	0.4399
	F				0.0531
	J				0.0234
TSC	S		0.5251	0.4703	0.3797
	F		0.4732	0.1682	0.0712
	J		0.2485	0.0791	0.0271
VAS	S	0.8116	0.5589	0.4698	0.4121
	F			0.1518	0.0564
	J			0.0713	0.0232
Mean	S	0.6525	0.5245	0.4448	0.3542
	F		0.2275	0.1104	0.0537
	J		0.1209	0.0492	0.0191
SE	S	0.1590	0.0184	0.0223	0.0215
	F		0.0831	0.0207	0.0053
	J		0.0442	0.0107	0.0022
ASU	S	0.9290	0.5343	0.4663	0.3714
	F	0.4035	0.2202	0.1079	0.0531
	J	0.3749	0.1177	0.0503	0.0197

Subjects were tested using Gábor patterns at four different Michelson contrasts. To estimate spatial extent entropy (S^2^), the spatial frequency was kept constant at 0.4 cycles/degree while the spatial standard deviation was varied around 1 deg. To estimate spatial frequency entropy (F), the spatial frequency was varied around 0.4 cycles/degree while the spatial standard deviation was kept constant at 1 deg. Joint entropy (J) was obtained by multiplying the spatial frequency entropy times the square root of the spatial extent entropy to account for the use of Gábor stimuli comprising 2D Gaussian envelopes and 1D sine wave gratings (J = F x S). For some subjects and stimulus conditions it was not possible to provide a good fitting to the data points (empty cells). However, for the ASU estimates, all data points were taken in consideration.

**Table 2:** Results for all seven subjects tested, means and standard errors of the means for the sample, as well as estimate results for an “average subject” (ASU; see text for details).

Subject		Contrast			
		2%	5%	10%	100%
BDG	S	0.4147	0.3706	0.3684	0.3465
	F	0.1696	0.1711	0.1405	0.1225
	J	0.0703	0.0634	0.0518	0.0424
DMR	S	0.5004	0.4407	0.4690	0.3362
	F	0.2359	0.1900	0.1452	0.1494
	J	0.1180	0.0838	0.0681	0.0502
GSS	S	0.3558	0.3164	0.3211	0.3003
	F	0.2459	0.1641	0.1404	0.1169
	J	0.0875	0.0519	0.0451	0.0351
IFA	S	0.3844	0.3556	0.2939	0.3152
	F	0.1683	0.1168	0.1342	0.0857
	J	0.0647	0.0415	0.0395	0.0270
RLB	S	0.3995	0.3725	0.3396	0.3512
	F	0.1277	0.1243	0.0824	0.1068
	J	0.0510	0.0463	0.0280	0.0375
TSC	S	0.4331	0.3999	0.3822	0.3878
	F	0.3320	0.2145	0.2046	0.2023
	J	0.1438	0.0858	0.0782	0.0784
VAS	S	0.4694	0.4778	0.4749	0.4735
	F	0.3441	0.1997	0.2220	0.1992
	J	0.1615	0.0954	0.1054	0.0943
Mean	S	0.4225	0.3905	0.3785	0.3587
	F	0.2319	0.1686	0.1528	0.1404
	J	0.0996	0.0669	0.0594	0.0522
SE	S	0.0188	0.0205	0.0265	0.0218
	F	0.0315	0.0140	0.0177	0.0172
	J	0.0160	0.0081	0.0100	0.0094
ASU	S	0.4316	0.3892	0.3753	0.3683
	F	0.2488	0.1765	0.1620	0.1526
	J	0.1074	0.0687	0.0608	0.0562

Spatial extent entropy (S^2^), spatial frequency entropy (F), and joint entropy (J) for 2 cycles/degree at four Michelson contrasts. All other details as in Table 1.

**Table 3:** Results for all seven subjects tested, means and standard errors of the means for the sample, as well as estimate results for an “average subject” (ASU; see text for details).

Subject		Contrast			
		2%	5%	10%	100%
BDG	S		0.5602	0.5090	0.3940
	F		4.7453	1.4481	0.8714
	J		2.6584	0.7370	0.3434
DMR	S			0.8057	0.5237
	F		2.4804	1.4608	2.9417
	J			1.1769	1.5407
GSS	S	0.6895	0.5681	0.5372	0.3825
	F	2.1067	1.9161	1.4722	1.1455
	J	1.4526	1.0886	0.7909	0.4381
IFA	S		0.5479	0.5896	0.3894
	F	2.1706	1.0703	0.9482	0.6533
	J		0.5864	0.5591	0.2544
RLB	S	0.6430	0.3950	0.5344	0.3450
	F	1.5419	1.6177	1.4003	0.8908
	J	0.9915	0.6390	0.7484	0.3073
TSC	S	0.7106	0.5831	0.5571	0.4930
	F		1.8379	1.9686	1.6421
	J		1.0717	1.0967	0.8095
VAS	S		0.5724	0.5446	0.4946
	F	1.8561	1.6510	1.5773	1.5500
	J		0.9451	0.8590	0.7666
Mean	S	0.6810	0.5378	0.5825	0.4317
	F	1.9188	2.1884	1.4679	1.3850
	J		1.1649	0.8526	0.6371
SE	S	0.0100	0.0290	0.0383	0.0264
	F	0.1428	0.4547	0.1132	0.2935
	J	0.2305	0.3112	0.0815	0.1721
ASU	S	0.8735	0.5972	0.5628	0.4393
	F	3.1003	1.9834	1.5403	1.2755
	J	2.7081	1.1845	0.8669	0.5604

Spatial extent entropy (S^2^), spatial frequency entropy (F), and joint entropy (J) for 10 cycles/degree at four Michelson contrasts. All other details as in Table 1.

**Figure 9:**
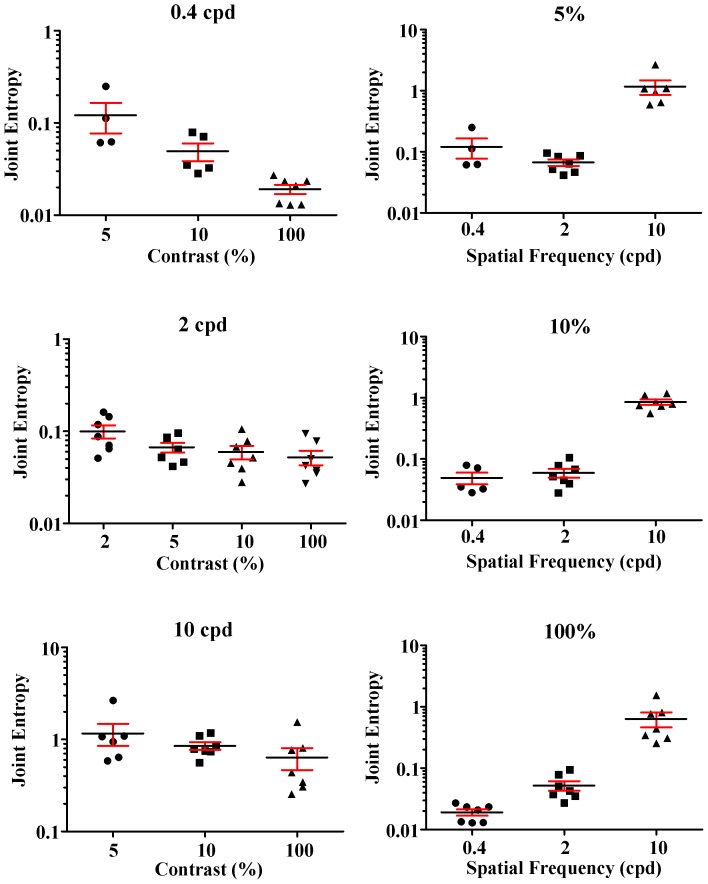
Statistical comparisons for the joint entropy measurements obtained from individual subjectspresented in Tables 1–3. Individual values for each subject, as well as means and standard errors of the means for the sample are plotted for different contrasts at 0.4, 2, and 10 cycles/degree (left panels, top to bottom) and for the three spatial frequencies at 5%, 10%, and 100% Michelson contrasts (right panels, top to bottom). Results for conditions with two or less measurements were not plotted (2% contrast at 0.4 and 10 cycles/degree). In all frequencies there was a trend for joint entropy to decrease when contrast was increased, but this reached the level of statistical significance only for the comparison between 5% and 100% contrasts (p<0.05; One-Way ANOVA, Tukey Multiple Comparison Test). For all contrasts, joint entropy was significantly lower at 0.4 and 2 cycles/degree when compared with 10 cycles/degree (p<0.05).

Figure 9 shows the statistical comparisons for the joint entropy measurements presented in Tables 1–3. Individual values for each subject, means, and standard errors of the means are plotted for different contrasts at 0.4, 2, and 10 cycles/degree (left panels, top to bottom) and for the three spatial frequencies at 5%, 10%, and 100% Michelson contrasts (right panels, top to bottom). In all spatial frequencies there was a trend for joint entropy to decrease when contrast was increased, but this reached the level of statistical significance only for comparisons between 5% and 100% contrasts (p<0.05; One-Way ANOVA, TukeyMultiple Comparison Test). For all contrasts, joint entropy was significantly lower at 0.4 and 2 cycles/degree when compared with 10 cycles/degree (p<0.05).

Tables 1–3 also show estimated values for an “average subject” (ASU subject), which were obtained by taken in consideration all data points from all subjects. In this regard, joint entropy values for the ASU subject could have differed from the joint entropy mean values obtained from the individual values for each subject. This was not generally the case as it can be seen in Tables 1–3 (see further below for details): for the majority of stimulus conditions,the mean values were similar to ASU values. Table 4 summarizes the results for the ASU subject.

**Table 4:** Estimate results for an “average subject” (ASU; see text for details).

Spatial Frequency		Contrast			
(cycles/degree)		2%	5%	10%	100%
0.4	S	0.9290	0.5343	0.4663	0.3714
	F	0.4035	0.2202	0.1079	0.0531
	J	0.3749	0.1177	0.0503	0.0197
2	S	0.4316	0.3892	0.3753	0.3683
	F	0.2488	0.1765	0.1620	0.1526
	J	0.1074	0.0687	0.0608	0.0562
10	S	0.8735	0.5972	0.5628	0.4393
	F	3.1003	1.9834	1.5403	1.2755
	J	2.7081	1.1845	0.8669	0.5604

One-dimensional entropies for the domain of space (S) an d spatial frequency (F), as well as the joint entropy (J) for 0.4, 2, and 10 cycles/degree. Estimates were based on data collected from seven subjects which were tested using Gábor patterns at four different Michelson contrasts.

**Figure 10:**
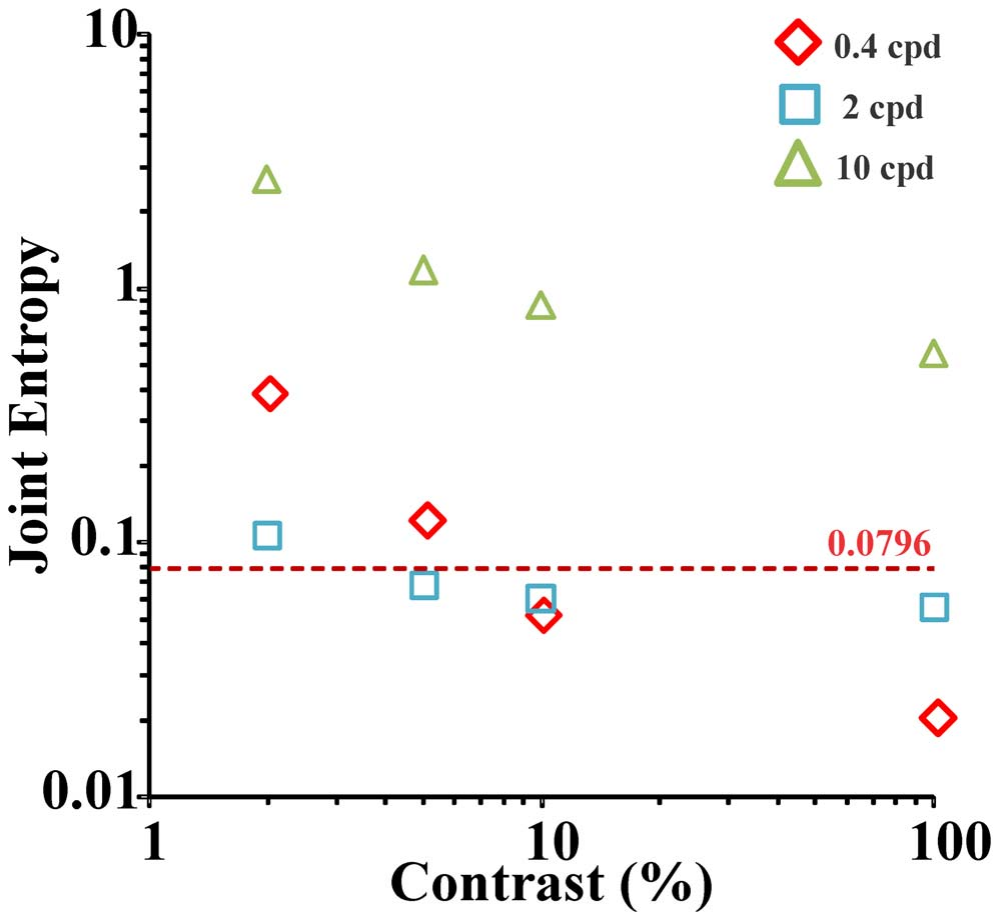
ASU (“average subject”) spatial and spatial frequency joint entropy as a function of Michelson contrast for three different spatial frequencies: 0.4, 2, and 10 cycles/degree (diamonds, square, and triangles, respectively). The dashed line represents the theoretical minimum for the 1D joint entropy of a system comprising only linear interactions between its subsystems. Using standard deviation as the entropy parameter and cycles/degree as spatial frequency metrics, the joint entropy minimum corresponds to 1/4π or 0.0796, and can only be attained by the product of the joint entropies for a Gábor function and its Fourier transform [14], [15], [22]. For all spatial frequencies, the joint entropy was higher at low contrasts and decreased when contrast was raised. At low and intermediate spatial frequencies and high contrasts, joint entropy reached levels below the minimum, an effect particularly pronounced at 0.4 cycles/degree and high contrast. This effect is suggestive that non-linear interactions between two or more visual mechanisms occur at these ranges of contrast and spatial frequency (see text for details).

Figure 10 shows plots of the “average subject” spatial and spatial frequency joint entropy as a function of Michelson contrast for 0.4, 2, and 10 cycles/degree (diamonds, square, and triangles, respectively). The minimum theoretical for the 1D joint entropy of a system comprising only linear interactions between its subsystems, using standard deviation as entropy measurement and cycles/degree as the metrics for spatial frequency, corresponds to 1/4π or 0.0796[14], [15], [21], [22]and it is indicated by a dashed line in the Figure 10. At all spatial frequencies, the joint entropy highest value was observed at the lowest contrast tested (2%) and then decreased when contrast was raised. At low contrast, the smallest value for joint entropy occurred at intermediate spatial frequency, 2 cpd, a frequency located in the region of human peak contrast sensitivity. This region of the spatial frequency domain (∼2–4 cpd) is supposed to contain the most important periodicities for visual behavior,especially face recognition [23]. Also, it is at these intermediate spatial frequencies that it is more clearly demonstrable the interaction of two or more visual pathways at high contrast levels and certain stimulus conditions(1 Hz square-wave contrast-reversal sine-wave gratings, transient VECP recording [13]; 10 Hzsine-wave contrast-reversal sine-wave gratings, steady-state sweep VECP recording[24];VECP elicited by sinusoidal gratings controlled by pseudo-random stimulation [25].

At intermediate spatial frequency (2 cpd), when contrast was raised, the joint entropy decreased to values below the theoretical minimum and remained low for the remained of the contrast range. This is consistent with the interaction of two or more visual pathways at this range of intermediate spatial frequencies and intermediate and high contrast levels [13].

At low spatial frequency (0.4 cpd),when contrast was raised, the joint entropy decreased at a faster rate and more pronouncedly than at intermediate or high spatial frequencies. The joint entropy remained below the theoretical minimum and at 100% contrast reached the lowest values for all combinations of spatial frequency and contrast that were tested.

At high spatial frequency(10 cpd), the joint entropy decreased at rate similar to intermediate spatial frequencies, but its values remained higher than at intermediate and low spatial frequencies and well above the theoretical minimum at all contrasts.

**Figure 11:**
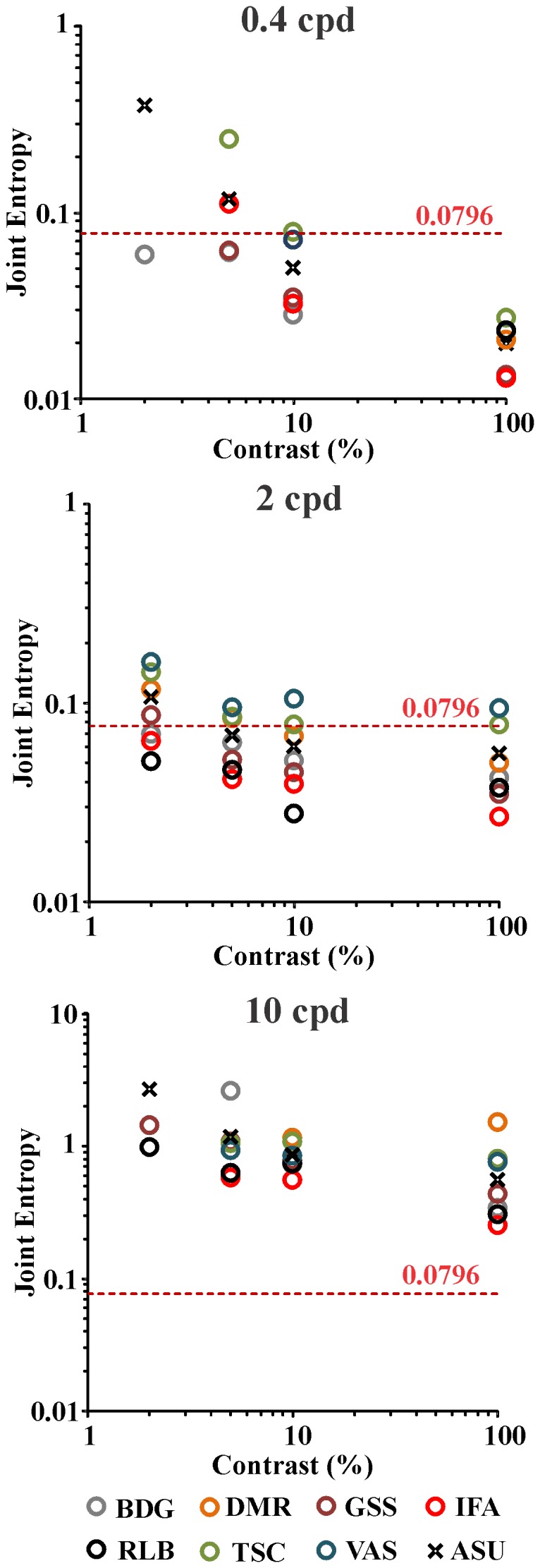
Spatial and spatial frequency joint entropy as a function of Michelson contrast for seven different subjects (circles) and for the “average subject” (crosses). From top to bottom the results for 0.4, 2, and 10/degree are presented, respectively. The dashed line represents the theoretical minimum for the 1D joint entropy of a system comprising only linear interactions between its subsystems, 0.0796. For most of cases, 10 out 12 conditions, the joint entropy for the “average subject” fells in the range of results for individual subjects. The two exceptions were conditions where only one or two individual results were obtainable by data fitting. Nevertheless, all data were used to estimate ASU results. For all subjects and spatial frequencies, the joint entropy was higher at low contrasts and decreased when contrast was raised. At low and intermediate spatial frequencies and high contrasts, joint entropy reached levels below the theoretical minimum, suggesting non-linear interactions between two or more visual mechanisms (see text for details).

In Figure 11 we compared the joint entropy values for the “average subject” (crosses) with those for individual subjects (circles). In most cases, the “average subject” values fell on the range of individual values with the exception of 2% contrast at 0.4 cpd and 10 cpd, where data fitting was only possible in one subject and two subjects, respectively.

## Discussion

### Joint entropy for space and spatial frequency at different spatial frequencies and contrasts

In this work, we used psychophysics methods to evaluate the visual system joint entropy in the domains of space and spatial frequency – two domains related by the Fourier transform – at a range of contrast levels.At all spatial frequencies, joint entropy decreased when stimulus contrast was raised. At low and intermediate spatial frequencies, 0.4 and 2 cpd, and high contrasts, 10% and 100%, joint entropy became smaller than the theoretical minimum for a system comprising only linear interactions between its subsystems, 1/4π or 0.0796 for the metric that was used[15].This finding suggeststhe presence of non-linear interactions of two or more visual mechanisms at these contrast levels. At high spatial frequency, 10 cpd, joint entropy remained above the theoretical minimum across the entire contrast range.

The decrease of joint entropy with the increase of stimulus contrast at all spatial frequencies is by itself suggestive that complex, non linear interactions between subsystems that input to the high levels of the visual system are at work. This suggestion becomesstronger at intermediate spatial frequencies (2 cpd) and low spatial frequency (0.4 cpd) once at these spatial frequencies joint entropy attained values below the theoretical minimum.

### Non linear interactions in the visual system

Non linear interactions might already be present at the level of subcortical neurons that inputto the primary visual cortex [26]and/or they might arise when two pathways interact in the visual cortex itself (see below the comment on the experiment by Palmer, Jones, Stepnoski [27]).

Kremers et al.have described a new type of contrast dependent nonlinear interaction between receptive center and surround that was present in all neuronal classes of primate lateral geniculate nucleus, including M and P cells, what was well correlated with results of psychophysics experiments performed using similar stimulus condition [26].The perception of flicker strength in a center stimulus was influenced by the relative phase of modulation in a surround stimulus and the response amplitudes of LGN neurons depended in a similar way on the relative phase between the modulation in the center and surround stimuli [26]. The contrast in the surround stimulus also had a similar effect both on the psychophysical and physiological data. The similarities between the psychophysical and physiological results suggested that the physiological basis of the perceived flicker strength in the center stimulus was already present in the retino-geniculate pathway [26]. In additional psychophysical experiments, Teixeira et al. isolated and investigated the subcortical and cortical lateral interactions involved in flicker perception using center and surround presented monoptically or dichopticallyand concluded that both subcortical and cortical lateral interactions modulated flicker perception [28].

### Convergence of visual pathways at higher levels of the visual system

It has been proposed, using computational arguments, that for the performance of many tasks, higher levels of the visual system need access to information from both the M and P pathways, performing some kind of concurrent processing [29]. Similar arguments can be considered when attempting to map the M and P pathways in the dorsal and ventral cortical streams both originating in early visual areas but terminating in posterior parietal or inferior temporal cortical regions, and when testing whether recognition of some stimuli relies on dorsal-ventral integration of information[30]. Previously we have argued that M and P cells perform simultaneous and overlapping analyses of the visual field using different strategies to minimize entropy[9]–[11]. This would enable higher order visual neurons [31]–[34]to combine M and P inputs in different ways, and could explain why M and P inputs need to converge at the high levels of the visual system after being kept separate at the subcortical levels[11], [35]. For instance, it has been shown in the cat that joint entropy of simple cortical cells for spatial and spatial frequency domains is smaller than the minimum predictable from Linear System Analysis [27]. Similarly to what we proposed for the primate visual system, the findings of Palmer et al. [27] could be attained in the cat visual system by a non-linear combination of information gathered by the alpha and beta cell pathwaysconverging at the visual cortex level [9].

### M and P pathways at the cortical level

This work tested the hypothesis thatnon-linear interactions at higher levels of the visual system might provide the system with the ability to perform different visual tasks with different contributions of its diverse visual pathways [11], [35]. Visual pathways such as the M and P arise from the same patch of retina and are kept separate until they reach the primary visual cortex entrance layers – 4Cα and 4Cβ for the M and P pathways, respectively [36]. At this level, a proportion of cortical cells exhibit a mix of M and P properties especially in the central zone of layer 4C [37]–[39]. Others neurons respectively located at the top and bottom regions of layer 4C have properties that are more pure representative of the M and P pathways [38].

Upstream to the primary visual cortex, two main cortical pathways convey visual information through a series of visual areas, the dorsal and ventral pathways. Different hypotheses have attributed specific properties and functional meanings to these pathways, such as object position and shape[40], movement and colour[41], and action and perception[42], respectively.In spite of demonstrations that the M and P pathways exhibit some degree of segregation of their cortico-cortical connections and that upstream cortical neurons also exhibit some degree of specificity for M or P receptive field properties [41], [43]–[45], generally it is hard to map either anatomically or physiologically the connections and response properties of M and P cells in an one to one basis onto the dorsal and ventral cortical pathways, suggesting that a certain degree of mixing M and P input do exist in these cortical pathways.

The results of this work suggest that visual system discrimination in the domain of space and spatial frequency requires non-linear interactions of different subsystems at the visual cortex level. The M and P pathways are well placed subsystems both in anatomical and physiological terms to represent the main source of these non-linearities at the cortex level.

### Future developments of this work

We are now investigating space and spatial frequency joint entropy of the human visual system by using psychometric functions obtained from discrimination of chromatic Gábor functions and comparing the results with those obtained with achromatic stimuli described in this paper[46]. In addition, we are combining temporal and spatial stimulus properties to measure the 6D joint entropy of the visual system in the domains of time, temporal frequency, 2D space, and 2D spatial frequency.

## Appendix

### Joint entropy theoretical minimum

The joint entropy or joint uncertainty theoretical minimum [14], [15] originated from the physical constraint to simultaneously increase precision in Fourier related domains [21]. This uncertainty principle was enunciated in 1925 by Norbert Wiener, during a Göttingen lecture, stating that a pair of transforms cannot both be very small; originated the famous physical demonstration in 1927 by Werner Heisenberg about the impossibility of specifying simultaneously the position and the momentum of an electron within an atom; and corresponds to the Pauli proposition in 1928 that the less the uncertainty for the square module of a function, the greater the uncertainty for the square module of its Fourier transform is, and conversely [22]. A demonstration for the existence of this minimum and its value can be found in several works using the Schwarz inequality [14], [21].

The square of entropy or uncertainty in the one dimensional space domain, *x*, or variance, is estimated by using the second momentum 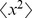 energy distribution of the complex signal 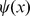 centered in its first momentum 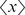 or centroid, the region where the function is more concentrated [21]. The first momentum is
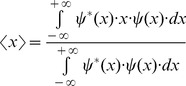
(2)While the second momentum is
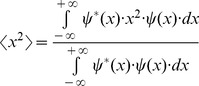
(3)The square of entropy or uncertainty centered in the first momentum is
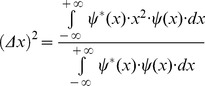
(4)The related domain of this complex signal 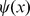 can be found by estimating its Fourier transform
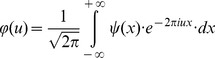
(5)This corresponds to the spectral function 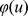 in the one dimensional spatial frequency domain, *u*. It is similarly possible to estimate the square of entropy or uncertainty in the spatial frequency domain using the following expression also centered in the first momentum:
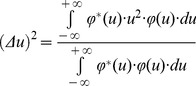
(6)By using the equations provided by Gábor [14],
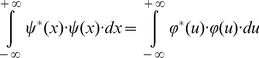
(7)

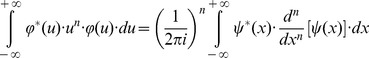
(8)

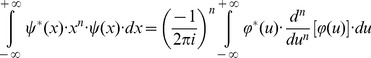
(9)and writing the square of spatial frequency entropy (Equation 6) in terms of its correlation in space (Equation 8), it follows that
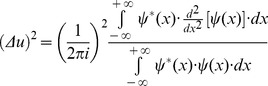
(10)Now, integrating by parts the numerator of Equation 10 and considering that the function 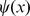 belongs to the Hilbert space, it is obtained
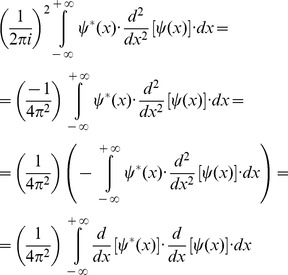
(11)Thus, it is possible now to substitute the result (Equation 11) in the numerator of the spatial frequency entropy square (Equation 10)
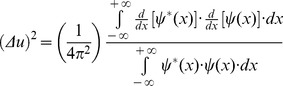
(12)Then, it is possible to multiply the two square entropies (Equations 4 and 12) and to estimate the joint entropy for the domains of space and spatial frequency:
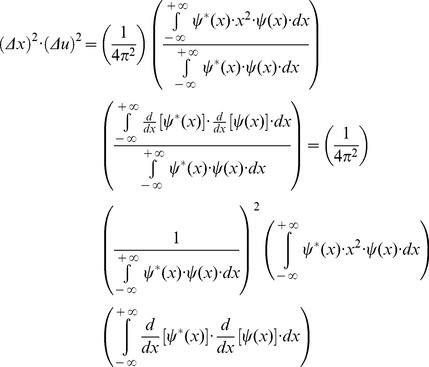
(13)The Schwarz inequality can then be used to show that the Equation 13 numeratorscan be related to the following [21]:
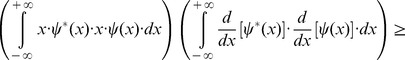


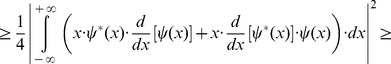


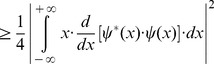
(14)Then, replacing the Inequality 14 result in the Equation 13 numerator, the product of the two entropy squares can be written as an inequality
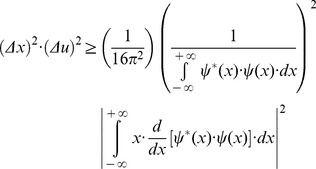
(15)Since 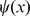 belongs to the Hilbert space and integrating by parts the square module from Equation 15 numerator, it follows that
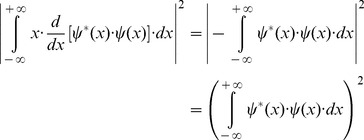
(16)and substituting the Equation 16 result in the joint entropy inequality (Inequality 15)
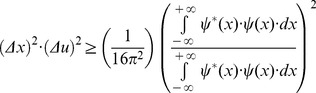
(17)the theoretical minimum cannot be less than 1/4π or 0.0796 (Inequality 18):
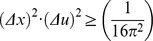


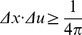
(18)


### Physical interpretation of entropy

Entropy is a large encompassing concept that is studied in theoretical thermodynamics and information theory, and it is related to very practical issues that arise from experiments in physics, chemistry, and biology. Entropy is defined in classical thermodynamics in terms of macroscopic measurements, while in information theoryis a measure of the uncertainty in a random variable. In classical thermodynamics, entropy makes no reference to any probability distribution, which is central to the definition of entropy in information theory. Thus, there is no obvious link between the use of entropy in fields such as classical thermodynamics and information theory.

Jaynes proposed that the thermodynamic entropy could be interpreted as proportional to the amount of additionalinformation, as defined in the Shannoninformation theory, needed to define the detailed microscopic state of the system, and that is not communicated by a description solely in terms of the classical thermodynamics macroscopic variables, with the Boltzmann constant asthe proportionality constant [47]. Thus, adding heat to a system increases its thermodynamic entropy because it increases the number of possible microscopic states for the system, thus making any complete state description longer and its information entropy larger.

### Entropy and vision

Entropy measurements have many applications in the study of nervous system, including quantification of information transmission by trains of nervous impulses [48], specification of receptive field properties of cortical cells [15], [49], [50], extraction of information from neuronal populations [51], and identification of integrated processes to measure functional cortical clusters in the study of counsciousness[52].

To perform well in the visual world, the visual system has to provide good accuracy (measurement proximity to the true value) and good precision (measurement reproducibility). However, several fundamentalphysical constraints are detrimental to visual system performance. For instance, there are conflicting temporal and spatial requirements to be overcame which are critical at low levels of retinal illuminance and could potentially reduce both accuracy and precision in the analysis of retinal images and guidance of bodymovements. Vision is also limited by another fundamental physical constraint, that of attaining simultaneously high precision in two domains related by the Fourier transform such as space and spatial frequency or time and temporal frequency[14], [15], [21]. Once natural visual stimuli are simultaneously composed by spatial and temporal localized features, as well as spatial and temporal periodicities, the visual system has to achieve the best combination of precision in time, space, temporal frequency, and spatial frequency to perform a given task [11]. In this regard, entropy is an inverse measurement of precision, and it can be used as such to evaluate visual system performance in the perception of complex stimuli as Gábor functions.
